# Disrupted astrocyte-neuron glutamine-glutamate cycling in the medial prefrontal cortex contributes to depression-like behaviors

**DOI:** 10.7150/ijbs.123740

**Published:** 2026-01-01

**Authors:** Jae Soon Kang, Ji Hyeong Baek, Hyeongchan Park, Naveed Ur Rehman, Hye Jin Chung, Dong Kun Lee, Sekyung Oh, Hyun Joon Kim

**Affiliations:** 1 Department of Anatomy and Convergence Medical Sciences, College of Medicine, Institute of Medical Science, Tyrosine Peptide Multiuse Research Group, Anti-aging Bio Cell Factory Regional Leading Research Center, Gyeongsang National University, Jinju, 52727, Republic of Korea.; 2 College of Pharmacy and Research Institute of Pharmaceutical Sciences, Gyeongsang National University, Jinju, 52828, Republic of Korea.; 3 Department of Physiology and Convergence Medical Sciences, College of Medicine, Institute of Medical Science, Tyrosine Peptide Multiuse Research Group, Gyeongsang National University, Jinju, 52727, Republic of Korea.; 4 Department of Medical Science, College of Medicine, Catholic Kwandong University, Incheon 22711, Republic of Korea.

**Keywords:** glutamate-glutamine cycle, emotional behavior, glutamatergic homeostasis, depression, depression animal model, cell-specific conditional knockout

## Abstract

The regulation of the homeostasis of the glutamate (Glu)-glutamine (Gln) cycle within the medial prefrontal cortex (mPFC) has garnered substantial interest due to its essential role in maintaining normal emotional behaviors. Chronic stress possesses the potential to disrupt the homeostasis of the Glu-Gln cycle, thereby facilitating the onset of depressive behaviors. Nevertheless, the specific roles of individual components within the Glu-Gln cycle in relation to depression-related behaviors remain incompletely understood. This study aims to elucidate the specific roles of each component by implementing a cell- and region-specific conditional knockout (cKO) strategy. To achieve this goal, the genes encoding glutamine synthetase (GS), glutamate transporter 1 (GLT-1), and sodium-coupled neutral amino acid transporters, SNAT-3 and SNAT-5, were selectively ablated within astrocytes. In addition, the genes encoding SNAT-1 and SNAT-2 were specifically eliminated from glutamatergic neurons. A depressive phenotype was observed in the GS and GLT-1 cKO mice, correlated with increased levels of reactive oxygen/nitrogen species (ROS/RNS) within the mPFC, whereas a reduction in Glu-Gln concentrations was uniquely identified in the GS cKO mice. Conversely, mice with cKO for SNAT-1, SNAT-2, SNAT-3, or SNAT-5 neither exhibited observable depressive-like behaviors nor reduced Glu-Gln levels. However, the simultaneous inactivation of either SNAT-1/SNAT-2 or SNAT-3/SNAT-5 induced depressive-like behaviors and reduced Glu-Gln levels. No systemic stress response or inflammatory manifestations were detected in any of the cKO mice. Furthermore, administration of Gln, acknowledged for its antidepressant properties, to GS cKO mice led to the amelioration of both depressive-like behaviors and Glu-Gln concentrations. These findings elucidate distinct and synergistic roles for the components involved in the Glu-Gln cycle in upholding appropriate Glu-Gln levels and mitigating ROS/RNS within the mPFC. Additionally, our cKO mouse models prove to be valuable tools in researching depression, which may aid in the development of new antidepressant treatments.

## Introduction

Major depressive disorder (MDD) represents a prevalent yet severe psychiatric condition 1. Manifesting in about 6% of the adult population on an annual basis 2, MDD has a high risk of leading to suicide, with individuals diagnosed with MDD exhibiting a suicide risk approximately 20 times greater than that of the general population 3. Aside from the high risk of suicide, it is particularly concerning that MDD was identified as the second leading cause of years lived with disability (YLD) and accounted for the largest share of mental YLDs in 2021. Furthermore, from 2010 to 2021, the 'age-standardized disability-adjusted life-years' for MDD increased by 16.4% 4. Therefore, it is crucial to develop innovative antidepressant treatments that are based on a thorough understanding of its etiological mechanisms.

Recently, the glutamate (Glu) hypothesis has garnered significant attention to elucidate the mechanisms of MDD. The hypothesis posits that diminished glutamatergic activity within the medial prefrontal cortex (mPFC) directly influences depressive behaviors, substantiated by evidence derived from both preclinical and clinical investigations. Notably, reductions in the quantity of astrocytes and the concentrations of Glu and glutamine (Gln) have been observed in patients exhibiting suicidal tendencies or depression 4-6. The activity within the mPFC of individuals diagnosed with depression was diminished, and the volume of this region was likewise reduced 7, 8. Additionally, both the antidepressant esketamine and the dextromethorphan-bupropion combination, approved by the US FDA, function through a shared mechanism that involves the activation of glutamatergic signaling in the prefrontal cortex 9-11.

The concentrations of Glu-Gln and the glutamatergic activity predominantly rely on the Glu-Gln cycle, which is sustained between glutamatergic neurons and astrocytes 11, 12. Glutamatergic neurons release Glu for neurotransmission. After signaling, astrocytes reuptake Glu via glutamate transporter 1 (GLT-1) 13. Within the astrocyte, glutamine synthetase (GS) transforms Glu into Gln. Gln is then conveyed to presynaptic glutamatergic neurons through sodium-coupled neutral amino acid transporters (SNAT)-3 and -5 in astrocytes and SNAT-1 and -2 in neurons. Finally, Gln is reconverted into Glu by glutaminase for subsequent signaling. (Fig. S1A). Disturbance in the Glu-Gln cycle has been demonstrated to induce depressive behaviors in rodent models 14-17. Supplementation with Gln rescues the deficits of Glu-Gln, thereby enhancing glutamatergic neurotransmission and demonstrating anti-depressive effects in a chronic immobilization stress (CIS)-induced depression mouse model 18. Furthermore, recent findings propose that activating GS compromised by CIS may represent a novel therapeutic strategy for depression 19.

Numerous studies have suggested the critical importance of homeostasis in the Glu-Gln cycle to preserve emotionally healthy behaviors, as well as elucidating the role of each key component within the Glu-Gln cycle to comprehend the underlying etiological mechanisms of MDD. From these considerations, this study employed a cell-specific CRISPR/CAS9 system to selectively suppress the expression of principal proteins, including GS, GLT-1, SNAT-1, SNAT-2, SNAT-3, and SNAT-5, within the mPFC, followed by an evaluation of the behavioral outcomes resulting from the conditional knockout (cKO) of each gene (Fig. S1B). Additionally, through biochemical analysis of stress biomarkers and amino acids, the impact of each protein cKO was investigated. Consequently, the study confirmed the role of each protein in normal behavioral manifestations and identified crucial etiological alterations within the mPFC that may lead to depressive behaviors, information that holds significant implications for the diagnosis of MDD and the development of novel antidepressants.

## Materials and Methods

### Small guide RNA (sgRNA) and adeno-associated virus (AAV)

For cloning sgRNA, we first generated the AAV viral vector of serotype 2, called pX552ch, by replacing the coding sequence (CDS) of EGFP of pX552 (Addgene, Watertown, MA, USA) with the CDS of mCherry. Various sgRNA sequences were subsequently subcloned into the sgRNA replacement site of pX552ch by using the HiFi assembly method (New England Biolabs, Ipswich, MA, USA), and the resulting plasmid were verified by sequencing. Sequences and vector map are summarized in Supplement information (Fig. S2 and Table S1). Using the constructed vector, serotype 2 AAV particles were produced at the Korea Institute of Science and Technology.

### Animals

We purchased a male and female pair of Vglut2-IRES-Cre (Stock No: 016963) and Rosa26-LSL-Cas9 knockin (Stock No: 026175, CRISPR/CAS9-EGFP) mouse from Jackson Laboratory (Bar Harbor, ME, USA). By breeding the Vglut2-Cre and the CRISPR/CAS9-EGFP mice, the Vglut2-IRES-Cre::CRISPR/CAS9-EGFP mouse line was prepared. For glutamatergic neuron-specific cKO, AAV2-px552ch-sgSlc38a1and/or Slc38a2 were delivered into prelimbic cortex (anteroposterior +1.5, mediolateral ±0.3 and dorsoventral -2.5 mm) via stereotaxic surgery of Vglut2-IRES-Cre::CRISPR/CAS9-EGFP mice. For astrocyte specific cKO, CRISPR/CAS9-EGFP mice were injected with pAAV.GFAP.Cre.WPRE.hGH to enable astrocyte-specific activity of the sgRNA. In Vglut2-IRES-Cre::CRISPR/CAS9-EGFP mice, EGFP was observed throughout the brain sections, including the prelimbic cortex (Fig. S1C). In contrast, in CRISPR/CAS9-EGFP mice, EGFP was observed only in the virus-injected region (Fig. S1D). The mice were treated by the protocol of the Institute Animal Care and Use Committee of Gyeongsang National University (approval No. GNU-190325-M0019).

### Behavioral tests

Two weeks after stereotaxic surgery, the behavioral tests, including open field test (OFT), tail suspension test (TST) and sucrose preference test (SPT), were conducted as previously described 17. The mice movement was tracked using EthoVision XT software (Noldus, Wageningen, Netherlands).

### Blood and tissue sampling

Mice were CO_2_ gas-anesthetized and their blood and mPFC of brain were collected between 9 to 11 am. Plasma was isolated using K_3_EDTA vacutainer by centrifuging at 4 ºC. The PFC samples were homogenized in RIPA buffer by glass beads using Bullet Blender Tissue Homogenizer (Next Advance, Troy, NY, USA). The resultant lysates were collected by centrifugation and stored at -80 ºC until use.

### Corticosterone (CORT) and ROS/RNS measurement

Levels of corticosterone and ROS/RNS were quantified using Corticosterone ELISA Kit (Cayman, Ann Arbor, MI, USA) and OxiSelect™ *In vitro* ROS/RNS Assay Kit (Cell Biolabs, San Diego, CA, USA) according to the protocol provided from the manufacturers.

### Amino acid analysis

In plasma and mPFC, amino acids (Glu, Gln and γ-aminobutyric acid (GABA)) were measured using liquid chromatography-mass spectrometry as described in the previous study 17. Briefly, lysates and plasma were centrifuged, mixed with an internal standard (L-Glu-d5), and analyzed using an Agilent 6460 system (Agilent, Singapore) with a SeQuant ZIC®-HILIC column (2.1×100 mm, 3.5 μm, 100 Å). Separation was achieved with a gradient of 0.1% formic acid in water and acetonitrile, and detection of Glu, Gln, and GABA was performed in multiple reaction monitoring detection method.

### Immunohistochemistry

After cardiac perfusion, the brain was sectioned to a 40 μm thickness by a vibratome (VT1200, Leica Biosystems, Nussloch, Germany). The selected sections were blocked with 3% BSA in 1× phosphate-buffered saline with 0.1% Tween®20. Tissues were sequentially reacted to primary antibody and Alexa Fluor 594- or Alexa Fluor 680-conjugated secondary antibody (Thermo Fisher Scientific, Waltham, MA, USA). The fluorescent image was obtained using a confocal microscope (Olympus, Tokyo, Japan). The antibody information is as follows: GS (MAB302, Merck Millipore, Burlington, MA, USA), GLT-1 (AB1783, Merck Millipore), SNAT-1 (12039-1-AP, Proteintech, Rosemont, IL, USA), SNAT-2 (sc-514037, Santa Cruz Biotechnology, Dallas, TX, USA), SNAT-3 (14315-1-AP, Proteintech), and SNAT-5 (ab72717, Abcam, Cambridge, UK).

### Western blot analysis

After SDS-polyacrylamide gel electrophoresis (SDS-PAGE), the separated proteins were transferred to polyvinylidene difluoride membrane (Bio-Rad, Hercules, CA, USA). The membrane was then blocked in 5% skim milk (BD Bioscience, Franklin Lakes, NJ, USA), followed by incubation with primary antibodies: GluA1 (MA5-27694, ThermoFisher Scientific), GluA2 (32-0300, ThermoFisher Scientific), GluN2A (sc-390094, Santa Cruz Biotechnology), GluN2B (sc-365597, Santa Cruz Biotechnology), and β-actin (MA1-140, ThermoFisher Scientific) and subsequently with goat anti-mouse IgG (H+L) secondary antibody (31430, ThermoFisher Scientific). The membrane was then reacted with Pierce™ ECL Western Blotting Substrate (ThermoFisher Scientific). Images were obtained using an iBright™ CL1500 Image System (ThermoFisher Scientific) and analyzed using iBright Analysis Software (ThermoFisher Scientific).

### Spontaneous excitatory postsynaptic current measurement

Measurement of spontaneous excitatory postsynaptic current (sEPSC) in the mPFC was conducted as described in a previous study 19. The brain slice including prelimbic cortex area was carefully placed on a recording chamber superfused with artificial cerebrospinal fluid. To remove GABAergic currents, 100 μM picrotoxin or 10 μM 6-cyano-7-nitroqui-noxaline-2,3-dione and 50 μM D-amino-phosphovaleric acid were treated. The pipette solution was composed of 130 mM KCl, 5 mM CaCl_2_, 10 mM EGTA, 10 mM HEPES, 2 mM MgATP, 0.5 mM Na_2_GTP, and 5 mM phosphocreatine. The currents were recorded from glutamatergic neurons with EGFP (holding potential was -70 mV) using a MultiClamp 700B Amplifier (Molecular Devices) in the whole-cell configuration, and analyzed by Clampfit (Ver. 11.1, Molecular Devices) and MiniAnalysis (Ver. 6, Synaptosoft, Fort Lee, NJ, USA).

### Statistical analysis

All data were represented as mean ± standard error of the mean (SEM) and statistically analyzed by one-way analysis of variance (ANOVA) with post hoc test or Student′s t-test (*p < 0.05*) using GraphPad Prism 9 (GraphPad Software, La Jolla, CA, USA).

## Results

### Astrocyte-specific GS knockout (GS cKO) causes depressive-like behaviors with the reduction of Glu and Gln levels

**Figure 1:**
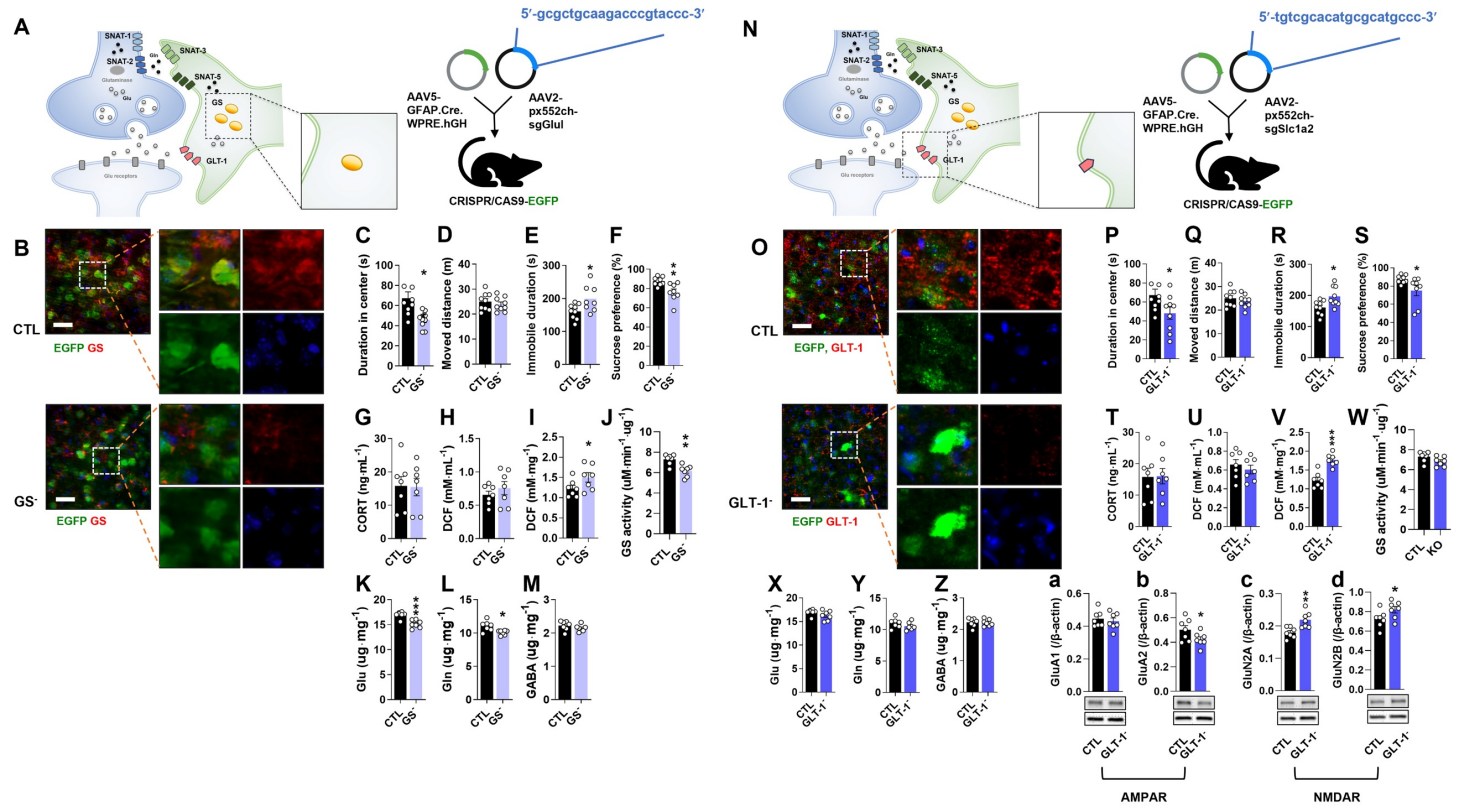
** Changes of phenotype in glutamine synthetase and glutamate transporter-1 (GLT-1) conditional knockout (cKO) mouse (GS^-^ and GLT-1^-^).** (A and N) Experimental scheme. The dotted and solid boxes indicate the target proteins of cKO and the expected changes, respectively. The two viruses for Cre recombinase and small guide RNA (sgRNA) were injected into the prelimbic cortex of CRISPR/CAS9-EGFP mice. The blue sequences indicate the sgRNA nucleotide sequences. (B and O) EGFP (green), GS or GLT-1 (red) signals in the prelimbic cortex. Blue signals are DAPI. Scale bars = 50 μm. (C and D, P and Q) Duration in center (C, t=3.151, df=16, *p=0.006*; P, t=2.126, df=16, *p=0.049*) and moved distance measured in open field test. (E and R) Immobile duration measured in tail suspension test (E, t=2.479, df=15, *p=0.026*; R, *t=2.784, df=16, p=0.013*). (F and S) Sucrose preference measured in sucrose preference test (F, t=3.298, df=15, *p=0.*005; S, t=2.348, df=15, *p=0.033*). (G and T) Plasma corticosterone (CORT) level. (H and I, U and V) Reactive oxygen/nitrogen species level in the plasma and medial prefrontal cortex (mPFC, I, t=1.941, df=12, *p=0.038*; V, t=5.400, df=12, *p<0.001*). DCF, 2', 7'-dichlorodihydrofluorescein. (J and W) GS activity in the mPFC (J, t=3.962, df=12, *p=0.002*). (K-M, X-Z) Glutamate, glutamine, and γ-aminobutyric acid (GABA) levels in mPFC (K-M, Glu, t=4.817, df=12, *p<0.001*; Gln, t=3.053, df=12, *p=0.010*). (a-d) Expression of GluA1 and GluA2 (b, t=1.959, df=12, *p=0.037*), subunits of α-amino-3-hydroxy-5-methyl-4-isoxazolepropionic acid receptor, GluN2A (c, t=3.509, df=12, *p=0.002*) and GluN2B (d, t=2.045, df=12, *p=0.032*), subunits of *N*-methyl-D-aspartate receptor in the mPFC of GLT-1 cKO mice. All data were represented as mean ± SEM and statistically analyzed by unpaired, two-tailed *t-test* (^*^p<0.05, ^**^p<0.01, and ^***^p<0.001).

To investigate the role of astrocytic GS in depression, GS cKO (GS^-^) mice were generated by co-injecting pAAV.GFAP.Cre.WPRE.hGH and pAAV2.pX552ch-sgGlul into CRISPR/CAS9-EGFP mice (Fig. 1A). Efficient GS knockout was confirmed by a marked reduction in GS immunoreactivity compared to the pAAV.GFAP.Cre.WPRE.hGH-injected group (CTL group) (Fig. 1B). Consistent with the hypothesis that GS dysfunction in astrocytes impairs glutamatergic homeostasis and induces depression-like phenotypes, GS cKO mice exhibited significant anxiety, helplessness, and anhedonia in the OFT, TST, and SPT, respectively (Fig. 1C-F). To assess if these behavioral abnormalities were linked with baseline levels of stress-sensitive marker expression, measurements of plasma corticosterone (CORT) and ROS/RNS levels were conducted. The deletion of GS did not lead to significant changes in either corticosterone or peripheral ROS/RNS levels (Fig. 1G, H). However, ROS/RNS levels in the mPFC were markedly increased (Fig. 1I). Furthermore, GS activity and the levels of Glu and Gln were significantly decreased in the mPFC of GS cKO mice, while GABA was not changed (Fig. 1J-M). These findings indicate that astrocytic GS dysfunction disrupts the Glu-Gln cycle and selectively elevates oxidative stress in the mPFC, which may underlie the observed depressive-like behaviors.

### Astrocyte-specific GLT-1 cKO causes depressive-like behaviors without changes in Glu and Gln levels

To examine the role of astrocytic GLT-1 in depression, GLT-1 cKO (GLT-1^-^) mice were generated by co-injecting pAAV.GFAP.Cre.WPRE.hGH and pAAV2.pX552ch-sgSlc1a2 into CRISPR/CAS9-EGFP mice (Fig. 1N). GLT-1 immunoreactivity was markedly reduced compared with controls (Fig. 1O). GLT-1 cKO mice displayed anxiety, helplessness, and anhedonia in the OFT, TST, and SPT, respectively (Fig. 1P-S). Although plasma CORT and ROS/RNS levels were unchanged (Fig. 1T, U), ROS/RNS in the mPFC increased significantly (Fig. 1V). GS activity and amino acid levels (Glu, Gln, GABA) were unaffected (Fig. 1W-Z). To see whether the increment of Glu in the synaptic cleft caused by GLT-1 cKO affects the expression of ionotropic glutamate receptors, the expression of α-amino-3-hydroxy-5-methyl-4-isoxazolepropionic acid (AMPA) 20 receptor subunit GluA1 and GluA2, and *N*-methyl-D-aspartate (NMDA) receptor subunit GluN2A and GluN2B were examined (Fig. 1a and b). Interestingly, GLT-1 deficiency altered glutamate receptor composition, decreasing the AMPA receptor subunit GluA2 and increasing NMDA receptor subunits GluN2A and GluN2B (Fig. 1a-d), suggesting a change of glutamate receptor-mediated excitatory signaling in the mPFC.

### Single knockout of SNAT-1 (SNAT-1 cKO) or SNAT-2 (SNAT-2 cKO) in glutamatergic neurons has no effect on emotional behaviors and Glu-Gln levels

**Figure 2:**
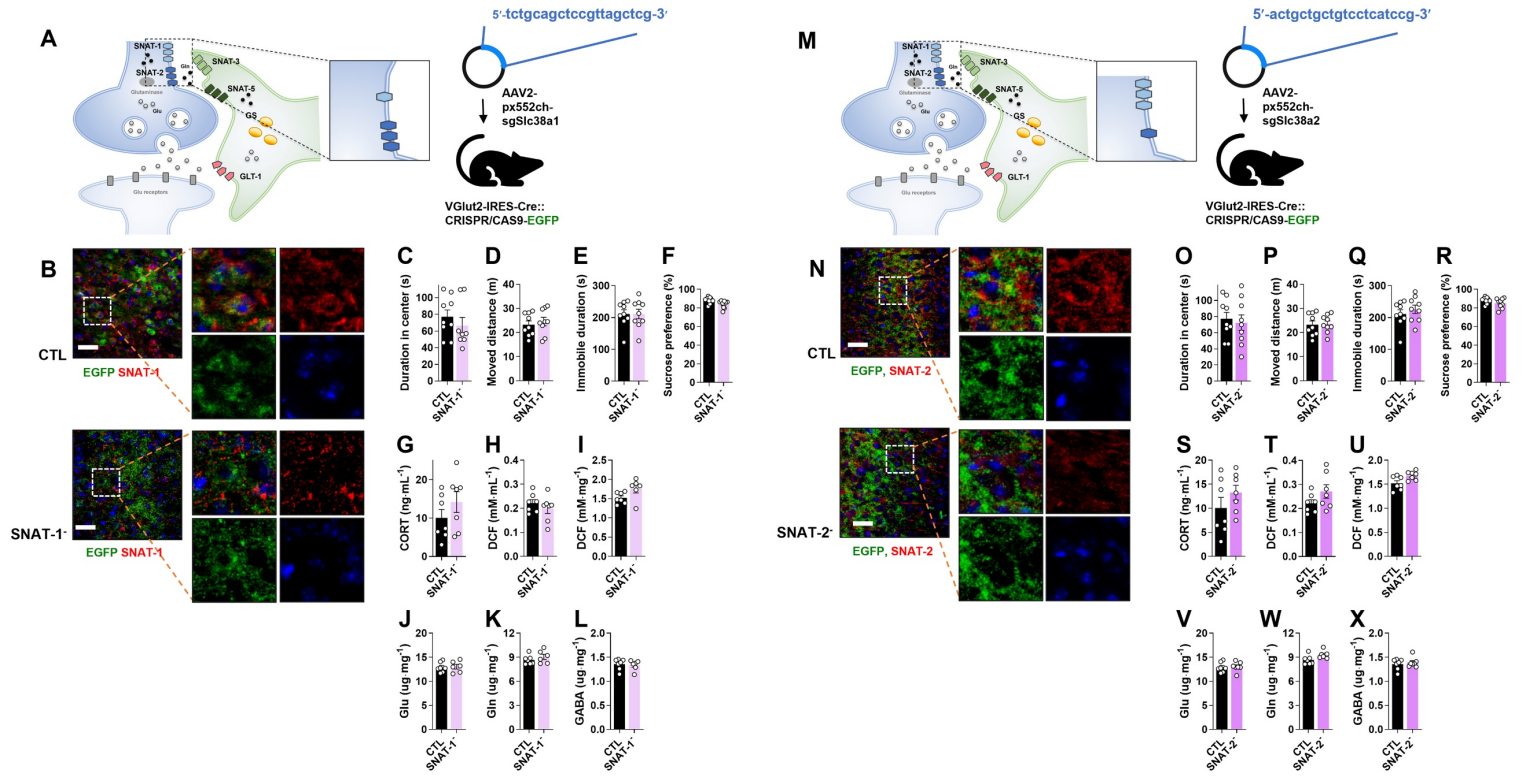
** Changes of phenotype in neuronal sodium-coupled neutral amino acid transporter-1 (SNAT-1) and SNAT-2 cKO mouse (SNAT-1^-^ and SNAT-2^-^).** (A and M) Experimental scheme. The dotted and solid boxes indicate the target proteins of cKO and the expected change, respectively. The virus for small guide RNA (sgRNA) of each gene was injected into the prelimbic cortex of Vglut2-IRES-Cre::CRISPR/CAS9-EGFP mice. The blue sequences indicate the sgRNA nucleotide sequences. (B and N) EGFP (green), SNAT-1 or SNAT-2 (red) signals in the prelimbic cortex. Blue signals are DAPI. Scale bars = 50 μm. (C and D, O and P) Duration in center and moved distance measured in the open field test. (E and Q) Immobile duration measured in the tail suspension test. (F and R) Sucrose preference measured in the sucrose preference test. (G and S) Plasma corticosterone (CORT) level. (H and I, T and U) Reactive oxygen/nitrogen species level in the plasma and medial prefrontal cortex (mPFC). DCF, 2', 7'-dichlorodihydrofluorescein. (J-L, V-X) Glutamate, glutamine, and γ-aminobutyric acid (GABA) levels in mPFC. All data were represented as mean ± SEM and statistically analyzed by unpaired, two-tailed *t-test* (*p < 0.05*).

To assess the role of neuronal glutamine transporters in depression, either SNAT-1 or SNAT-2 was selectively knocked out (SNAT-1 cKO and SNAT-2 cKO) in glutamatergic neurons by injecting pAAV2.pX552ch-sgSlc38a1 or pAAV2.pX552ch-sgSlc38a2 into Vglut2-IRES-Cre::CRISPR/CAS9-EGFP mice, respectively (Fig. 2A, M). Successful deletion of either SNAT-1 or SNAT-2 was confirmed by immunohistochemistry (Fig. 2B, N). Neither SNAT-1 nor SNAT-2 cKO mice exhibited depressive-like behaviors in the OFT, TST, or SPT (Fig. 2C-F, 2O-R). The levels of CORT, ROS/RNS, and amino acids (Glu, Gln, and GABA) were also unchanged (Fig. 2G-L, 2S-X).

### Astrocyte-specific SNAT-3 knockout (SNAT-3 cKO) evokes anxious behavior with the reduction of Glu and GABA levels

**Figure 3:**
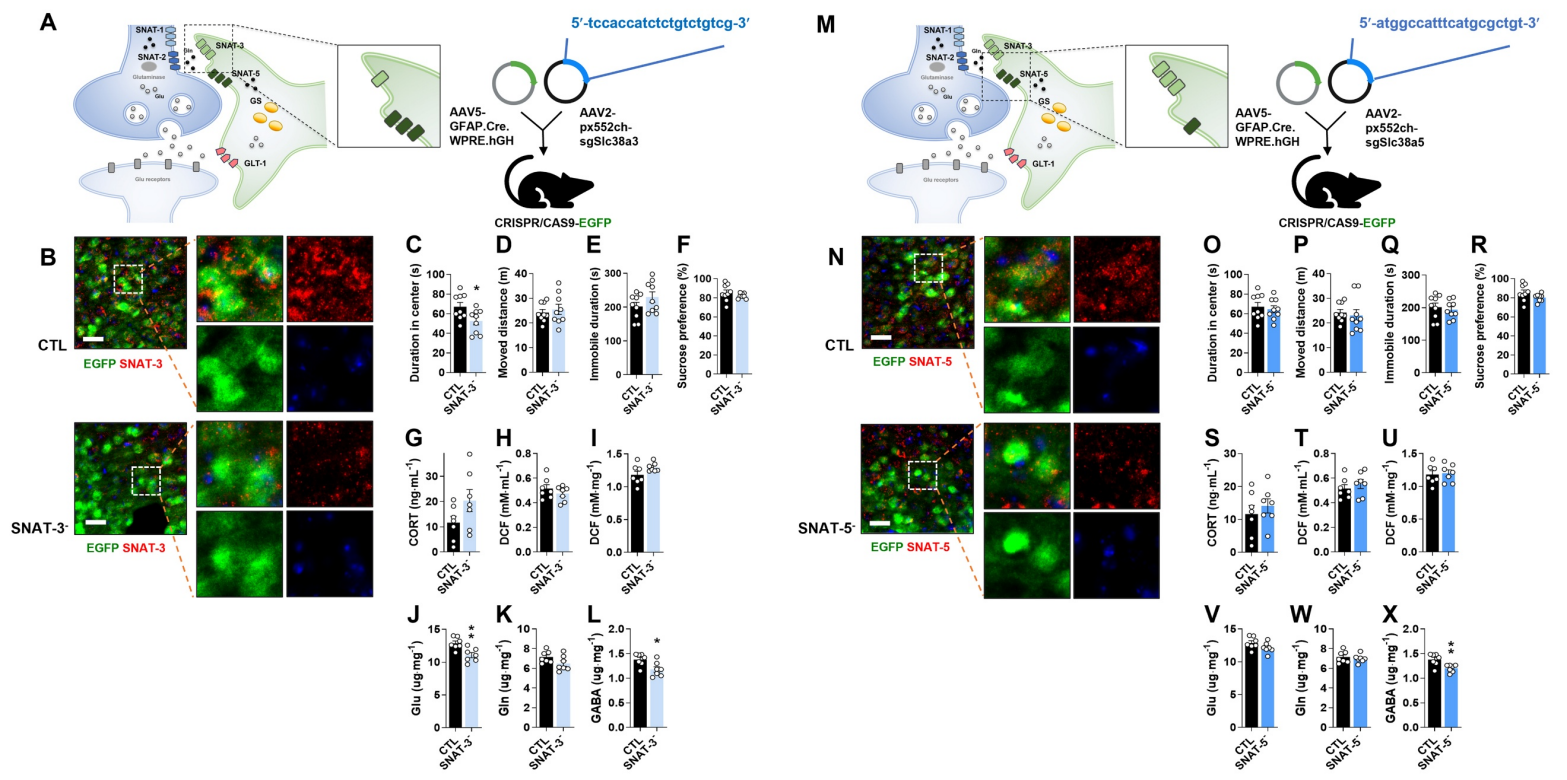
** Changes of phenotype in astrocytic sodium-coupled neutral amino acid transporter-3 (SNAT-3) and SNAT-5 conditional knockout (cKO) mouse (SNAT-3^-^ and SNAT-5^-^).** (A and M) Experimental scheme. The dotted and solid boxes indicate the target proteins of cKO and the expected change, respectively. The two viruses for Cre recombinase and small guide RNA (sgRNA) were inserted into the prelimbic cortex of CRISPR/CAS9-EGFP mice. The blue sequences indicate the sgRNA nucleotide sequences. (B and N) EGFP (green), SNAT-3 or SNAT-5 (red) signals in the prelimbic cortex. Blue signals are DAPI. Scale bars = 50 μm. (C and D, O and P) Duration in center (C, t=2.440, df=16, *p=0.*027) and moved distance measured in the open field test. (E and Q) Immobile duration measured in the tail suspension test. (F and R) Sucrose preference measured in the sucrose preference test. (G and S) Plasma corticosterone (CORT) level. (H and I, T and U) Reactive oxygen/nitrogen species level in the plasma and medial prefrontal cortex (mPFC). DCF, 2', 7'-dichlorodihydrofluorescein. (J-L, V-X) Glutamate (J, t=3.497, df=12, *p=0.004*), glutamine, and γ-aminobutyric acid (GABA, L, t=2.735, df=12, *p=0.018*; X, t=3.222, df=12, *p=0.007*) levels in mPFC. All data were represented as mean ± SEM and statistically analyzed by unpaired, two-tailed *t-test* (^*^p<0.05, ^**^p<0.01).

To investigate the role of astrocytic SNAT-3 in depression, pAAV.GFAP.Cre.WPRE.hGH and pAAV2.pX552ch-sgSlc38a3 were co-injected into CRISPR/CAS9-EGFP mice (Fig. 3A). SNAT-3 immunoreactivity was markedly reduced in the mPFC of SNAT-3 cKO mice (Fig. 3B). In behavioral tests, SNAT-3 cKO mice spent less time in the center zone during the OFT, indicating increased anxiety-like behavior, while total locomotor activity was not affected (Fig. 3C and D). Performance in the TST and SPT was unchanged (Fig. 3C-F). Plasma CORT and ROS/RNS levels showed no changes (Fig. 3G-I). Amino acid analysis revealed selective reductions in Glu and GABA (Fig. 3J-L). These results suggest that astrocytic SNAT-3 loss alters neurotransmitter balance without inducing overt depressive-like behaviors.

### Astrocyte-specific SNAT-5 knockout (SNAT-5 cKO) shows normal behaviors with only a decrease in GABA

To examine the role of astrocytic SNAT-5 in depression, pAAV.GFAP.Cre.WPRE.hGH and pAAV2.pX552ch-sgSlc38a5 were co-injected into the mPFC of CRISPR/CAS9-EGFP mice (Fig. 3M). SNAT-5 immunoreactivity was markedly reduced in the mPFC of SNAT-5 cKO mice (Fig. 3N). SNAT-5 cKO mice showed no abnormal behaviors in the OFT, TST, or SPT, and no significant changes were detected in plasma CORT, and ROS/RNS (Fig. 3O-U). Among amino acids, only GABA levels were decreased (Fig. 3X), suggesting a minor effect of astrocytic SNAT-5 loss on GABA-mediated inhibitory neurotransmission without behavioral consequences.

### Glutamatergic neuronal specific double knockout of SNAT-1 and SNAT-2 (SNAT-1/SNAT-2 dcKO) induces depressive-like behaviors with a decrement of Glu and Gln

**Figure 4:**
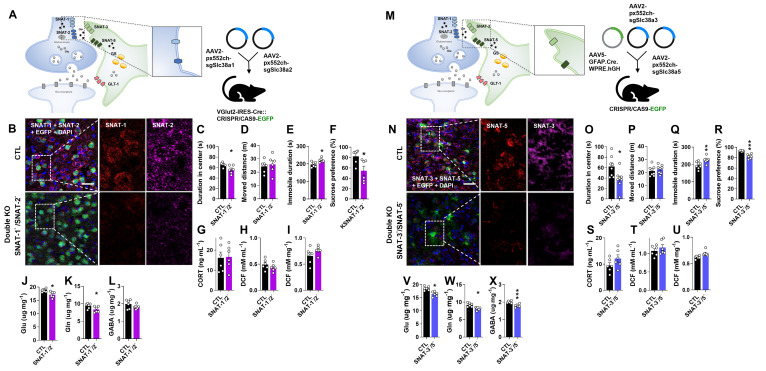
** Changes of phenotype in double conditional knockout (dcKO) of SNAT-1/SNAT-2 and SNAT-3/SNAT-5 mouse (SNAT-1^-^/SNAT-2^-^ and SNAT-3^-^/SNAT-5^-^).** (A and M) Experimental scheme. The dotted and solid boxes indicate the target proteins of dcKO and the expected change, respectively. For dcKO of neuronal SNATs (SNAT-1^-^/SNAT-2^-^), the two viruses with sgRNA were injected into prelimbic cortex of Vglut2-IRES-Cre::CRISPR/CAS9-EGFP mice. For dcKO of astrocytic SNATs (SNAT-3^-^/SNAT-5^-^), one virus for Cre recombinase and two viruses for sgRNA were inserted prelimbic cortex of CRISPR/CAS9-EGFP mice. (B and N) Green is for EGFP, red is for SNAT-1 or SNAT-5, and violet is for SNAT-2 or SNAT-3 in the prelimbic cortex. Blue is for DAPI. Scale bars = 50 μm. (C and D, O and P) Duration in center (C, t=2.636, df=10, *p=0.*025; O, t=2.604, df=14, *p=0.*021) and moved distance measured in open field test. (E and Q) Immobile duration (E, t=3.155, df=10, *p=0.010*; Q, t=3.160, df=14, *p=0.007*) measured in tail suspension test. (F and R) Sucrose preference (F, t=2.669, df=10, *p=0.0241*; R, t=7.196, df=14, *p<0.001*) measured in sucrose preference test. (G and S) Plasma corticosterone (CORT) level. (H and I, T and U) Reactive oxygen/nitrogen species level in the plasma and medial prefrontal cortex (mPFC). DCF, 2', 7'-dichlorodihydrofluorescein. (J-L, V-X) Glutamate (J, t=2.926, df=10, *p=0.015*; S, t=4.317, df=10, *p=0.002*), glutamine (K, t=2.200, df=10, *p=0.026*; T, t=3.790, df=10, *p=0.004*), and γ-aminobutyric acid (GABA, U, t=4.834, df=10, *p<0.001*) levels in mPFC. All data were represented as mean ± SEM and statistically analyzed by unpaired, two-tailed *t-test* (^*^p<0.05, ^**^p<0.01, and ^***^p<0.001).

To investigate the combined role of neuronal glutamine transporters, SNAT-1 and SNAT-2 double conditional knockout (SNAT-1/SNAT-2 dcKO) mice were generated by co-injecting pAAV2.pX552ch-sgSlc38a1 and pAAV2.pX552ch-sgSlc38a2 into Vglut2-IRES-Cre::CRISPR/CAS9-EGFP mice (Fig. 4A). Immunohistochemistry confirmed a marked reduction of both SNAT-1 (violet) and SNAT-2 (red) signals (Fig. 4B). Unlike the single knockouts (SNAT-1 cKO and SNAT-2 cKO), SNAT-1/SNAT-2 dcKO mice exhibited clear depressive phenotypes across all behavioral tests, including OFT, TST, and SPT (Fig. 4C-F). However, CORT and ROS/RNS levels remained unchanged in both plasma and mPFC (Fig. 4G-I). Amino acid analysis revealed significant reductions in Glu and Gln (Fig. 4J-L), suggesting that concurrent loss of neuronal SNAT-1 and SNAT-2 critically impairs the Glu-Gln cycle, leading to depressive-like phenotypes.

### Astrocyte-specific double knockout of SNAT-3 and SNAT-5 (SNAT-3/SNAT-5 dcKO) results in depressive-like behaviors and decrements of Glu, Gln, and GABA

To examine the combined roles of astrocytic glutamine transporters, SNAT-3 and SNAT-5 double conditional knockout (SNAT-3/SNT-5 dcKO) mice were generated by co-injecting pAAV.GFAP.Cre.WPRE.hGH, pAAV2.pX552ch-sgSlc38a3, and pAAV2.pX552ch-sgSlc38a5 into CRISPR/CAS9-EGFP mice (Fig. 4M). Immunohistochemistry confirmed markedly reduced SNAT-3 (violet) and SNAT-5 (red) signals in the mPFC (Fig. 4N). SNAT-3/SNAT-5 dcKO mice exhibited pronounced depressive behaviors across the OFT, TST, and SPT compared with CTL (Fig. 4O-R). However, CORT and ROS/RNS levels were unchanged (Fig. 4S-U). Notably, the concentrations of Glu, Gln, and GABA were all significantly decreased (Fig. 4V-X), suggesting that concurrent loss of SNAT-3 and SNAT-5 severely disrupts the Glu-Gln-GABA cycle, leading to depressive-like phenotypes.

### Gln supplementation ameliorates depressive-like behaviors and hypoactive glutamatergic neurotransmission in the mPFC of GS cKO

**Figure 5:**
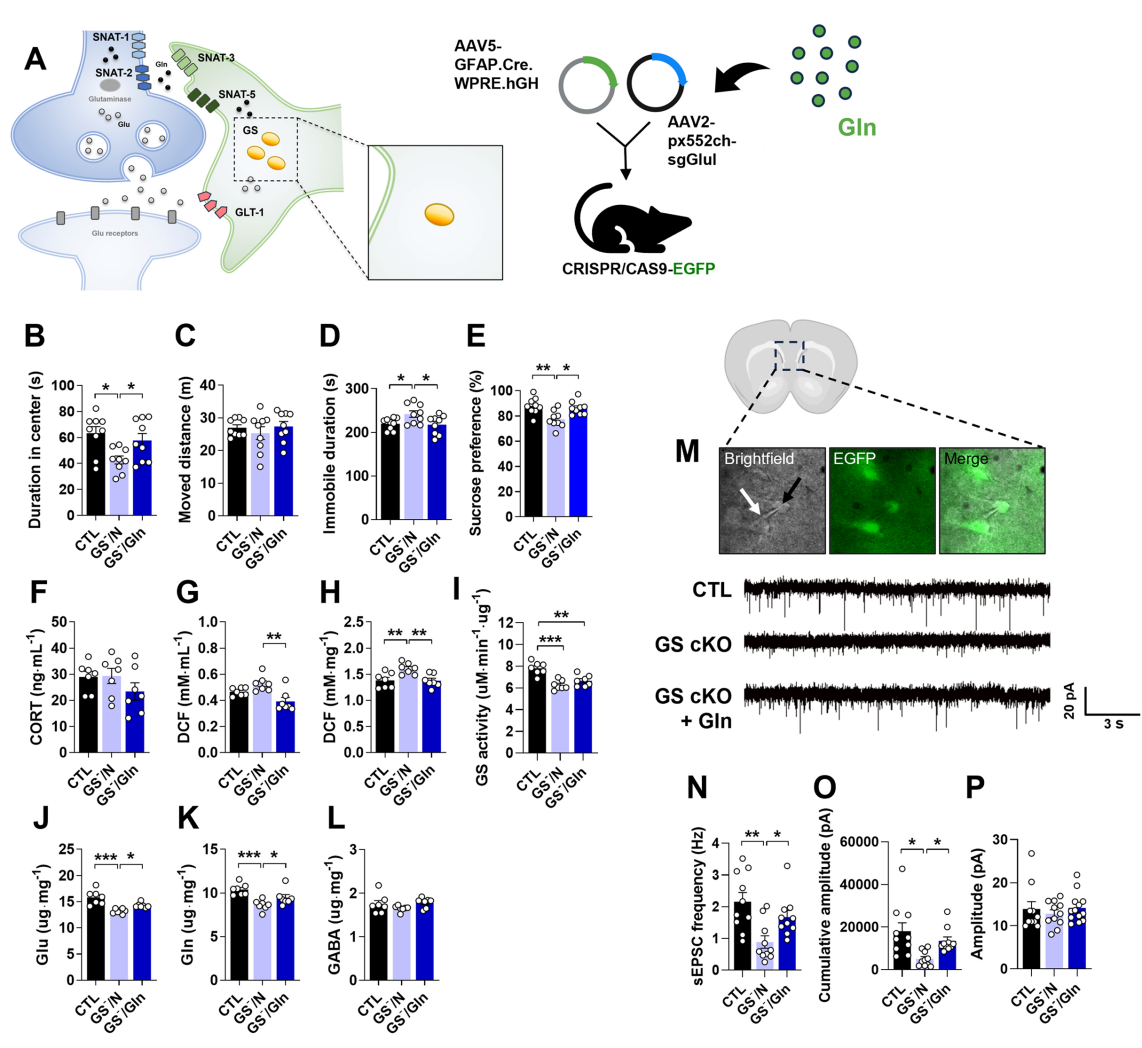
** Anti-depressive effects of Gln supplementation in GS cKO mouse.** (A) Experimental scheme. The dotted and solid boxes indicate the target proteins of cKO and the expected change, respectively. The two viruses for Cre recombinase and small guide RNA (sgRNA) were injected into the prelimbic cortex of CRISPR/CAS9-EGFP mice. Gln diet was provided to mice from 7 d before surgery to decapitation. (B and C) Duration in center (B, F_(2,24)_=5.349, *p=0.*012) and moved distance measured in the open field test. (D) Immobile duration (F_(2,24)_=4.128, *p=0.*029) measured in the tail suspension test. (E) Sucrose preference (F_(2,24)_=7.467, *p=0.003*) measured in the sucrose preference test. (F) Plasma corticosterone (CORT) level. (G and H) Reactive oxygen/nitrogen species level in plasma (F_(2,18)_=8.907, *p=0.002*) and medial prefrontal cortex (mPFC, F_(2,18)_=7.418, *p=0.005*). DCF, 2', 7'-dichlorodihydrofluorescein. (I) GS activity in the mPFC. (J-L) Glutamate (F_(2,18)_=15.74, *p<0.001*), glutamine (F_(2,18)_=10.34, *p=0.001*), and γ-aminobutyric acid (GABA) levels in mPFC. (M) Measuring of spontaneous excitatory postsynaptic current (sEPSC) using patch clamp probe (white arrow) in glutamatergic neuron (green, black arrow) of prelimbic cortex, and sEPSC recordings measured. (N-P) sEPSC frequency (F_(2,31)_=5.662, *p=0.008*), cumulative amplitude (F_(2,31)_=5.499, *p=0.009*), and average amplitude. All data were represented as mean ± SEM and statistically analyzed by one-way analysis of variance (ANOVA) with post hoc Dunnett′s Multiple Comparison Test (^*^p<0.05, ^**^p<0.01, and ^***^p<0.001).

The depressive-like behaviors and decreased Glu-Gln levels observed in GS cKO mice (Fig. 1A-M) appear to correspond with the effects noted in the mouse model of depression induced by CIS 17, 19. Given our previous findings that Gln exhibits an antidepressive effect in CIS-induced depressiveness, we examined whether Gln supplementation could ameliorate the deleterious effects of GS cKO. To this end, Gln was supplied to the GS cKO mice for one week before and two weeks after surgery (Fig. S1B). The GS cKO mice treated with Gln exhibited behaviors in the OFT, TST, and SPT that were similar to those observed in the CTL (Fig. 5B-E). This result provides strong evidence that Gln supplementation effectively reversed the depressive phenotype. Interestingly, the stress bio-marker CORT level remained indifferent among groups, including the Gln-treated GS cKO group (Fig. 5F). In GS cKO mice, the increased levels of ROS/RNS were decreased by Gln supplementation, bringing them back to CTL levels in both plasma and mPFC (Fig. 5G and H). Similar to its impact on the CIS-induced depression mouse model 18, Gln supplementation did not alter GS activity in GS cKO mice (Fig. 5I) but instead compensated for the Glu and Gln deficiencies, rescuing them to the CTL levels (Fig. 5J and K), indicating a recovery of the Glu-Gln balance. GABA level, however, showed no significant difference across the three groups (Fig. 5L). Given that the disturbance of Glu-Gln balance in the mPFC affects glutamatergic neurotransmission, we investigated sEPSC to assess synaptic function (Fig. 5M). The frequency of sEPSC, which was lower in GS cKO compared to CTL, returned to the CTL level after Gln supplementation (Fig. 5N). Moreover, in the GS cKO, the cumulative amplitude was reduced, but it was reversed by Gln supplementation (Fig. 5O). There was no difference in the average amplitude size among the three groups (Fig. 5P). These electrophysiological results indicate that Gln supplementation can preserve glutamatergic synaptic function in the mPFC by ammeliorating disrupted Glu-Gln homeostasis.

## Discussion

**Figure 6:**
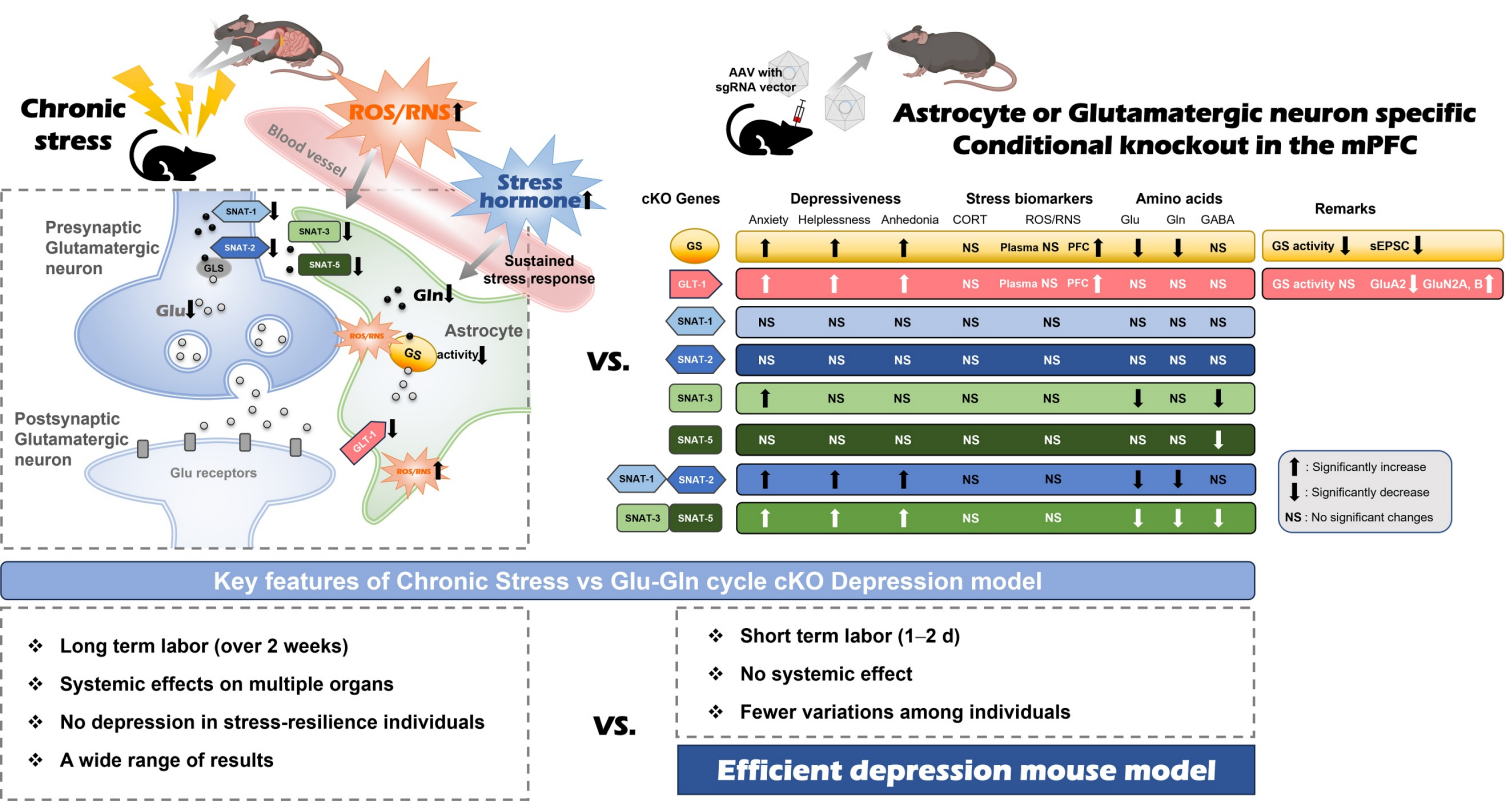
** Comparison between chronic immobilization stress (CIS)-induced depression model (left) and astrocyte- or glutamatergic neuron-specific conditional knockout (cKO) mouse (right).** The astrocyte or glutamatergic neuron-specific cKO mice have the advantages of a short labor time, no systematic effects, and fewer experimental variations among individuals compared to the CIS model, so it can be used as an effective depression animal model.

The importance of the glutamatergic system in the PFC has recently emerged, supported by a multitude of preclinical and clinical studies that reinforce the glutamate hypothesis 10, 14, 17, 21, 22. We previously reported a decrease in GS activity and a downregulation of protein expression associated with the Glu-Gln cycle, including GLT-1, SNAT-1, SNAT-2, SNAT-3, and SNAT-5, in the mPFC of mice with CIS-induced depression 14, 17, 18. The objective of this research was to elucidate the functions of these proteins in the manifestation of typical emotional behaviors and to pinpoint the critical factors that cause the transition from normal to depressive-like behaviors (Fig. 6).

Within the proteins associated with the Glu-Gln cycle, GS serves a critical function in the recycling process of Glu absorbed from the synaptic cleft. Our prior research indicated that the inhibition of GS or the ablation of astrocytes led to the manifestation of depressive behaviors 14. It was also demonstrated that the reduction of GS activity induced by CIS exerts a causative influence on depressive-like behaviors, with its activity being downregulated via tyrosine nitration by peroxynitrite, which is augmented by CIS 17, 19. Furthermore, CIS-induced depressive-like behaviors were ameliorated through Gln-supplementation or the process of denitration by means of tyrosine and tyrosyl-glutamine 17, 19. Consequently, we hypothesized that the abrogation of GS activity would manifest depressive phenotypes and a reduction in Glu-Gln concentrations within the mPFC. Our current research, employing the GS cKO model, has tested and confirmed this hypothesis to be the case. Alongside these phenotypes, we observed an elevation in ROS/RNS levels, which was associated with a reduction in GS activity. Although synaptic Glu-Gln cycling and neuronal activity are likely diminished in GS cKO mice, the astrocytic build-up of Glu and ammonium may lead to a metabolic burden and oxidative/nitrosative stress within astrocytes, a situation attributed to GS deficiency 23. The compartmentalized reaction, characterized by reduced neurotransmission along with heightened astrocytic metabolic stress, may explain the paradoxical increase in ROS/RNS levels. These alterations, instigated by GS cKO, consequently culminated in hypoactive glutamatergic neurotransmission as demonstrated in the CIS-induced depression mouse model 17, 19, which predominantly accounts for the manifestation of depressive-like behaviors. Hence, our results indicate that an unimpaired GS function may be essential for preserving normal emotional behaviors.

Our findings further imply the potential applicability of GS cKO as an alternative to the CIS-induced depression mouse. To evaluate this potential, Gln was administered to GS cKO mice due to the known antidepressant properties of Gln 17, 18. Gln-administered GS cKO mice exhibited emotional behaviors and sEPSC that were comparable to the control group. Gln supplementation resulted in a reduction of ROS/RNS levels. Notably, there was no alteration in GS activity, which is consistent with our prior findings 17. We thus propose that GS cKO may serve as a model for the development of novel antidepressants aimed at mitigating depressive behaviors induced by chronic stress.

Gln, which is converted from Glu in astrocytes, is transported to glutamatergic neurons. This is mediated by the SNAT family. Consequently, it has been proposed that the expression levels of the SNAT family are significantly linked to depressive behaviors, with a decrease in their expression closely associated with a higher incidence of suicidal behavior 24. Inhibition of neuronal glutamine transporters resulted in decreased levels of Glu-Gln in the mPFC and manifested depressive-like behaviors 14, and a reduction of their expression was observed in CIS-induced depressive mice 18. Furthermore, the reduction in the expression of SNAT-3 and SNAT-5 in astrocytes was also validated 18. Nevertheless, there is a paucity of information regarding the role of these SNATs in the regulation of normal behaviors. To our knowledge, this study is the first to demonstrate neuron-specific cKO of SNAT-1 or SNAT-2 in the mPFC, along with astrocyte-specific cKO of SNAT-3 or SNAT-5. Through this methodology, we confirmed the distinct roles of these in maintaining the homeostasis of the Glu-Gln cycle and the expression of normal behaviors. It is notable that no substantial alteration was observed in the single cKO of SNAT-1 or SNAT-2. On the other hand, the dcKO of SNAT-1/SNAT-2 resulted in depressive-like behavior and decreased levels of Glu-Gln, which is consistent with the results observed when their function is inhibited 14, 25. The findings suggest that the two transporters fulfill complementary roles in the uptake of glutamine into glutamatergic neurons. Conversely, anxiety-like behavior and a reduction in Glu and GABA levels were observed with SNAT-3 cKO. While depressive-like behavior was absent in SNAT-5 cKO, a reduction in GABA levels was noted. The phenotypes in the single cKO of SNAT-3 or SNAT-5 appeared less pronounced compared to the pronounced depressiveness and diminished Glu-Gln levels in SNAT-3/SNAT-5 dcKO. This observation implies that the roles of SNAT-3 and SNAT-5 appear to be somewhat asymmetrically arranged compared to the compensatory roles of SNAT-1 and SNAT-2 in neurons.

The tripartite synapse consists of presynaptic and postsynaptic neurons, in addition to an astrocyte, with the potential for the neurons to be either glutamatergic or GABAergic in nature 26, 27. The cycle involving astrocytes and GABAergic neurons is referred to as the GABA-Gln cycle. Within this cycle, GABA is absorbed by GABA transporter-1 located in astrocytes and subsequently converted into Gln, which is then conveyed to GABAergic neurons 26. Notably, a reduction in GABA levels was observed in all SNAT-3, SNAT-5 cKO, and SNAT-3/SNAT-5 dcKO conditions, whereas the GABA level remained unchanged in other cKOs with normal SNAT-3 and SNAT-5 expression (Fig. 6). These findings suggest that adequate exportation of Gln from astrocytes represents a crucial regulatory point for maintaining GABA levels in the brain, achievable solely via the function of both SNAT-3 and SNAT-5.

Recent studies have proposed that the antidepressant esketamine obstructs NMDA receptors on GABAergic neurons, leading to the disinhibition of glutamatergic neurons, thereby inducing a rapid increase in glutamatergic signaling within the mPFC 28-30. However, if patients exhibit insufficient functionality of SNAT-3 or SNAT-5 within the mPFC, the resultant decrease in GABA levels would lead to a diminished inhibitory effect of GABAergic neurons on glutamatergic neurons. Consequently, the patient may exhibit an inadequate response to esketamine 31. In such a case, alternative therapeutic approaches need to be considered, and our SNAT-3 and SNAT-5 cKO models may serve as appropriate animal models for the development of novel antidepressants to offset esketamine.

Incomplete clearance of glutamate by astrocytes has been noted in various psychiatric disorders 32-34, and the reduced expression of GLT-1 in the brain has been posited to be intricately associated with depression 18, 35, 36. From this perspective, the manifestation of depressive symptoms in GLT-1 cKO was anticipated. Nevertheless, the phenotype exhibited by GLT-1 cKO presents as particularly intriguing when juxtaposed with our prior hypothesis, which suggested that the augmentation of ROS/RNS in the PFC would mitigate GS activity through tyrosine nitration, thereby leading to reduced levels of Glu-Gln and diminished glutamatergic signaling 17, 19. GLT-1 cKO exhibits depressive phenotypes accompanied by an elevation of ROS/RNS in the mPFC, while the activity of GS and the levels of Glu-Gln appear to remain unchanged. Consequently, the depressive manifestations observed in GLT-1 cKO may be induced by alternative mechanisms. GLT-1 is predominantly expressed in astrocytes and at reduced levels within excitatory presynaptic terminals 13, 37. The ablation of GLT-1 from astrocytes across the entire brain resulted in merely a 15% decrease in Glu uptake into forebrain synaptosomes. Conversely, the neuronal deletion of GLT-1 led to a reduction of synaptosomal Glu uptake to 40% of that observed in the wild type 37. It is plausible that the GLT-1 cKO retains a considerable proportion (~80%) of Glu synaptosomes as found in CTL, potentially reliant on the Glu-Gln cycle facilitated by the Glu aspartate transporter (GLAST) in astrocytes and GLT-1 in presynaptic neurons. Consequently, no significant alterations in the Glu-Gln levels have been observed in GLT-1 cKO in this study.

While a substantial portion of Glu in the synaptic cleft is cleared by GLAST and neuronal GLT-1, there remains an excess of Glu within the synaptic cleft, influencing the postsynaptic neuronal excitability. Should the postsynaptic neurons incur damage due to excitotoxicity, abnormal behaviors would be expected during behavioral assessments. However, no abnormal behaviors are observed within their home environments or during behavioral tests, as indicated by consistent movement distances in the OFT. Previous research has also indicated that susceptibility to excitotoxicity is predominantly contingent upon GLT-1 in presynaptic terminals rather than in astrocytes 37, 38. Hence, GLT-1 cKO appears unaffected by the augmented excitatory Glu stimulation resulting from an increased Glu within the synaptic cleft.

Excitotoxicity requires the excessive activation of Glu receptors on postsynaptic neurons. Conversely, desensitization of Glu receptors can serve as a protective mechanism against excitotoxicity and may function as a feedback system to mitigate the adverse effects of excessive Glu 39, 40. Glu initially activates AMPA receptors, given their primary responsibility for rapid neurotransmission, and then activation of NMDA receptors occurs 41-44. In this investigation, the expression of GluA2 is diminished by GLT-1 cKO, which appears to play a role in attenuating Glu-induced rapid depolarization. Notably, the expression of GluN2A and GluN2B subunits is elevated. Under normal physiological conditions, an augmented ligand does not necessitate an increased number of receptors if the affinity or sensitivity remains unaltered. Nonetheless, should the receptors undergo desensitization, the neuron might require a surge in receptor quantity to preserve its responsiveness to stimuli 45. The NMDA receptor initiates a calcium influx upon activation, which subsequently stimulates the generation of superoxide within postsynaptic neurons 46. GluN2B can produce nitric oxide (NO) through its interaction with postsynaptic density protein 95 and neuronal nitric oxide synthase 47. NO reacts with superoxide to form peroxynitrite, which in turn leads to the nitration of tyrosine or the nitrosylation of cysteine residues within proteins, resulting in dysfunction 19, 48. Indeed, both nitration and nitrosylation are proposed as therapeutic targets to ameliorate excitotoxic neuronal damage in severe excitotoxicity cases, such as stroke or epilepsy 48, 49. The observed increase in ROS/RNS in GLT-1 cKO appears to manifest within postsynaptic neurons rather than astrocytes, as evidenced by the unchanged activity of GS. The escalation in ROS/RNS may be precipitated by NMDA receptor signaling in postsynaptic neurons, which leads to the desensitization of NMDA receptors through nitration and nitrosylation, thereby conferring protection against Glu-induced excitotoxicity. These processes may subsequently result in diminished glutamatergic signaling, which could underlie depressive behaviors in GLT-1 cKO. Similar to dysfunction states of SNAT-3 and SNAT-5, esketamine may not exert a therapeutic effect in patients lacking fully functional GLT-1 on astrocytes, due to the presence of desensitized NMDA receptors in the mPFC.

Animal models designed to study chronic stress-induced depression in numerous laboratory settings exhibit several constraints, such as prolonged labor requirements, unexpected extensive systemic effects, and markedly variable results attributable to the differing stress sensitivities of individual animals. However, the cKO models in this study can mitigate these limitations as they present certain advantages over chronic stress-induced depression models (Fig. 6). The cKOs targeting the Glu-Gln cycle proteins exhibit localized effects and can be produced using a relatively straightforward technique within a comparatively brief timeframe, which facilitates the generation of consistent phenotypes. Furthermore, the current study revealed the role for each Glu-Gln cycle component in depression pathogenesis. Consequently, it is anticipated that the cKOs of Glu-Gln cycle proteins may be beneficial as model systems in the testing of novel antidepressants.

## Supplementary Material

Supplementary figures and table.

## Abbreviations

MDD: major depressive disorder
YLD: years lived with disability
Glu: glutamate
Gln: glutamine
mPFC: medial prefrontal cortex
GLT-1: glutamate transporter 1
GS: glutamine synthetase
SNAT: sodium-coupled neutral amino acid transporter
CIS: chronic immobilization stress
cKO: conditional knockout
sgRNA: small guide RNA
AAV: adeno-associated virus
OFT: open field test
TST: tail suspension test
SPT: sucrose preference test
CORT: corticosterone
GABA: γ-aminobutyric acid
PAGE: polyacrylamide gel electrophoresis
sEPSC: spontaneous excitatory postsynaptic current
AMPA: α-amino-3-hydroxy-5-methyl-4-isoxazolepropionic acid
NDMA: *N*-methyl-D-aspartate
NO: nitric oxide
