# Supplementary material for: Disrupted astrocyte-neuron glutamine-glutamate cycling in the medial prefrontal cortex contributes to depression-like behaviors
Source: Int J Biol Sci. 2026 Jan 1;22(2):553–65. doi: 10.7150/ijbs.123740 (PMC12780845; doi:10.7150/ijbs.123740)
Supplement: Supplementary file 1 — Supplementary figures and table. [file ijbsv22p0553s1.pdf]

Disrupted astrocyte-neuron glutamine-glutamate cycling in the medial prefrontal cortex contributes to depressive-like behaviors

Jae Soon Kang[1], Ji Hyeong Baek[1], Hyeongchan Park[1], Naveed Ur Rehman[2], Hye Jin Chung[2], Dong Kun Lee[3], Sekyung Oh[4,*] and Hyun Joon Kim[1,*]

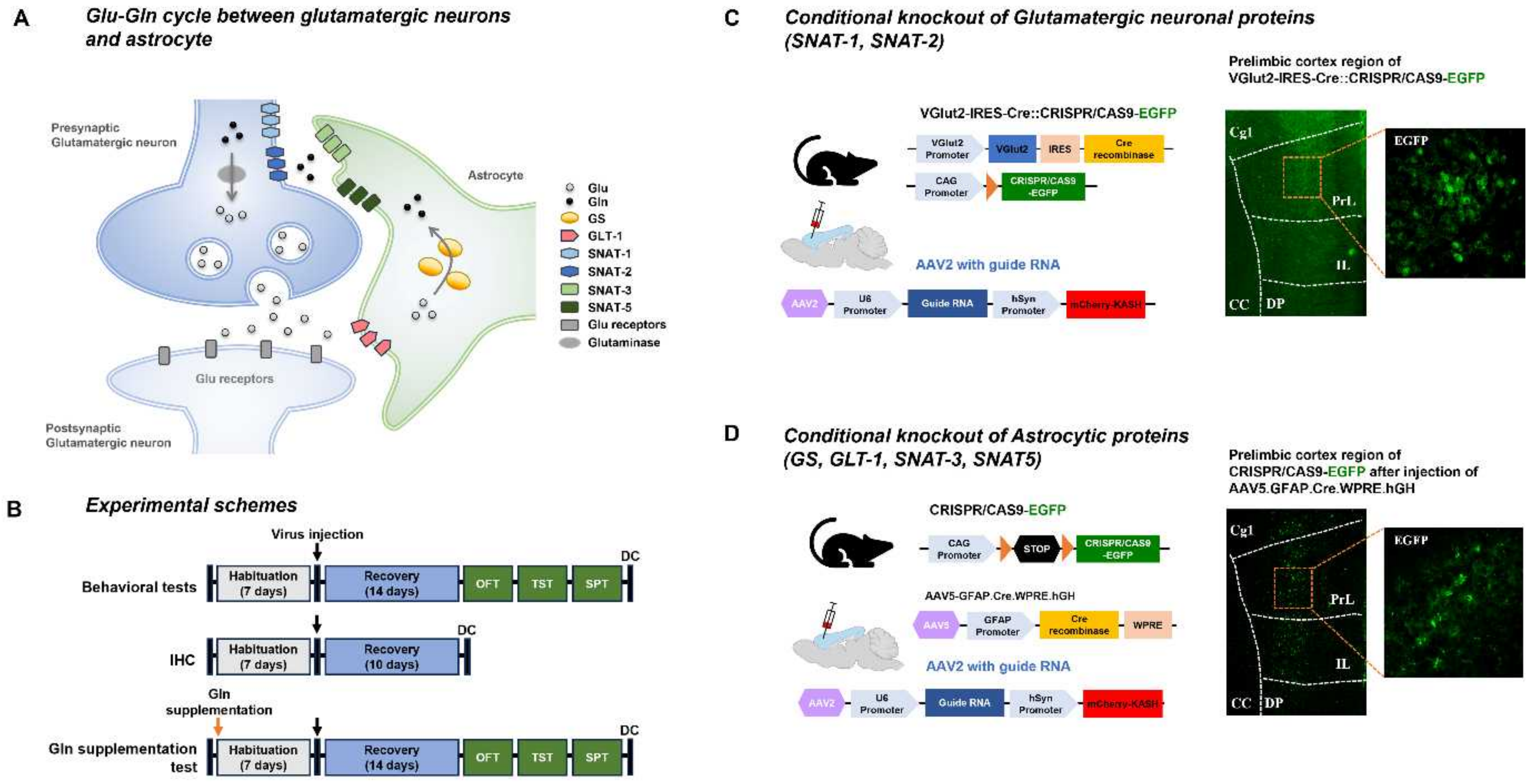

Fig. S1. Overall information of experiments in the present study. (A) Schematic drawing of target components investigated in this study and their positions in the Glu-Gln cycle between glutamatergic neurons and astrocyte, (B) experimental timeline of each experiment, (C) Schematic diagrams of Vglut2-IRES-Cre::CRISPR/CAS9-EGFP and AAV2 with small guide RNA, and the representative EFGP signals of the prelimbic cortex after infection. (D) Schematic diagrams of CRISPR/CAS9-EGFP, AAV5-GFAP.Cre.WPRE.hGH, AAV2 with small guide RNA, and the representative EGFP signals of the prelimbic cortex after infection.

Table S1. Sequences of each small guide RNA used in this study

**Gene**	**SgRNA (5¢-3¢)**	**Orientation**
GluI	gcgctgcaagacccgtaccc	forward
Slc1a2	tgtcgcacatgcgcatgccc	reverse
Slc38a1	tctgcagctccgttagctcg	reverse
Slc38a2	actgctgctgtcctcatccg	reverse
Slc38a3	tccaccatctctgtctgtcg	reverse
Slc38a5	atggccatttcatgcgctgt	forward

Fig. S2. Information of vector map used in this study (see the next page)

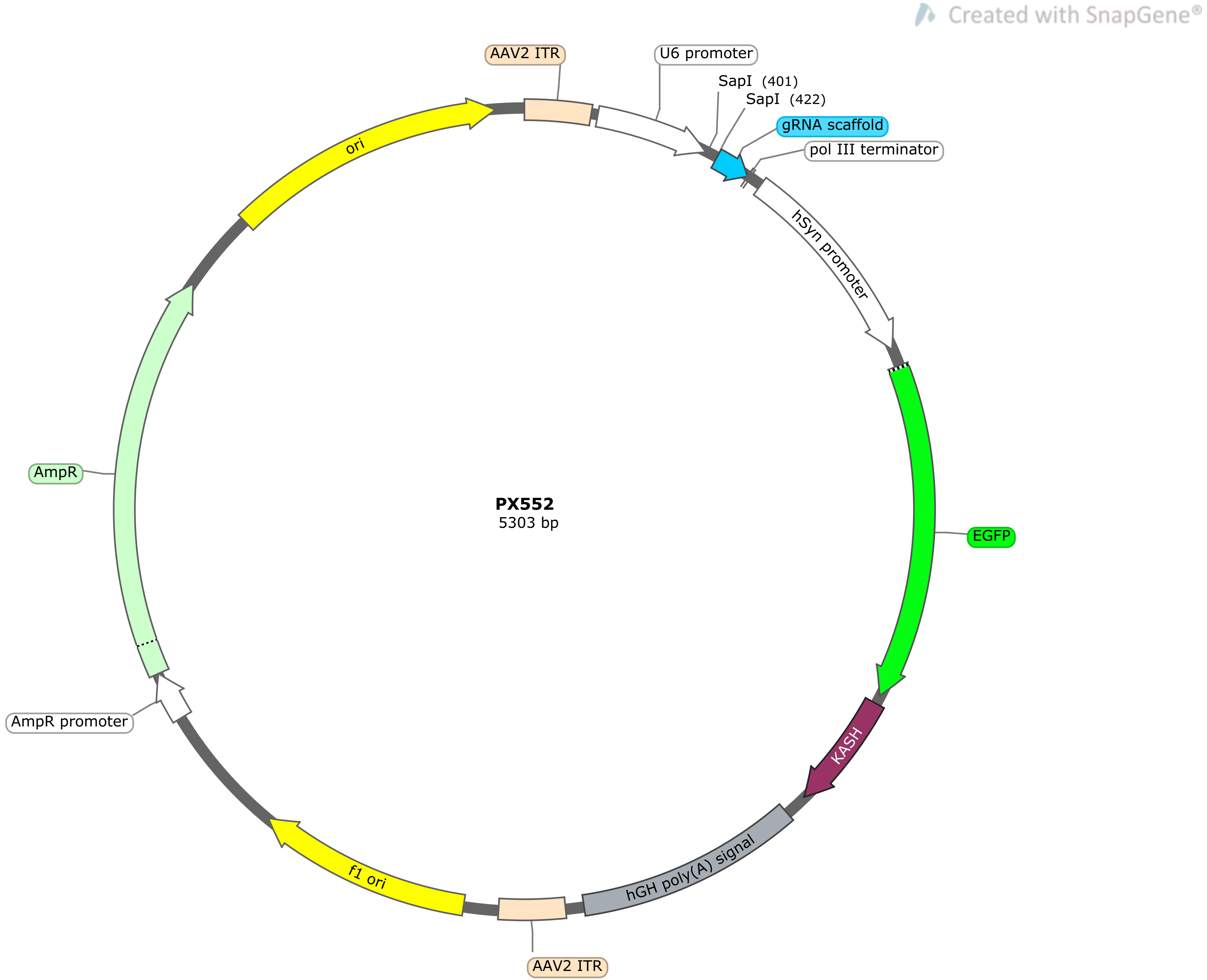
Created with SnapGene®
PX552
5303 bp
AAV2 ITR
U6 promoter
SapI (401)
SapI (422)
gRNA scaffold
pol III terminator
hSyn promoter
EGFP
KASH
hGH poly(A) signal
AAV2 ITR
f1 ori
AmpR promoter
AmpR
ori

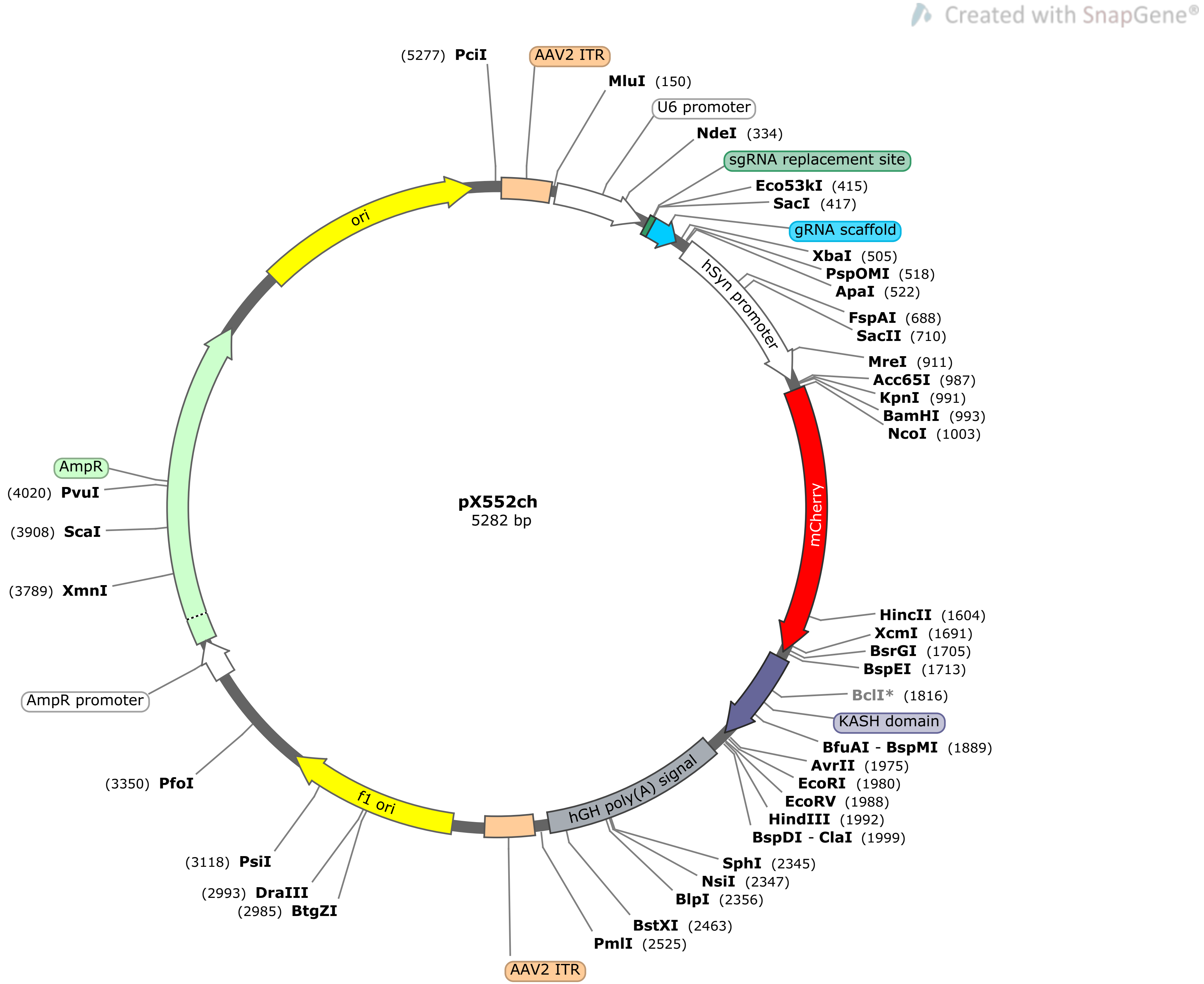

Created with SnapGene®
pX552ch
5282 bp
(5277) PciI
AAV2 ITR
MluI (150)
U6 promoter
NdeI (334)
sgRNA replacement site
Eco53kI (415)
SacI (417)
gRNA scaffold
XbaI (505)
PspOMI (518)
ApaI (522)
FspAI (688)
SacII (710)
hSyn promoter
MreI (911)
Acc65I (987)
KpnI (991)
BamHI (993)
NcoI (1003)
mCherry
HincII (1604)
XcmI (1691)
BsrGI (1705)
BspEI (1713)
BclI* (1816)
KASH domain
BfuAI - BspMI (1889)
AvrII (1975)
EcoRI (1980)
EcoRV (1988)
HindIII (1992)
BspDI - ClaI (1999)
hGH poly(A) signal
SphI (2345)
NsiI (2347)
BlpI (2356)
BstXI (2463)
PmlI (2525)
AAV2 ITR
f1 ori
(3118) PsiI
(2993) DraIII
(2985) BtgZI
(3350) PfoI
AmpR promoter
(3789) XmnI
(3908) ScaI
(4020) PvuI
AmpR
ori

Created with SnapGene®

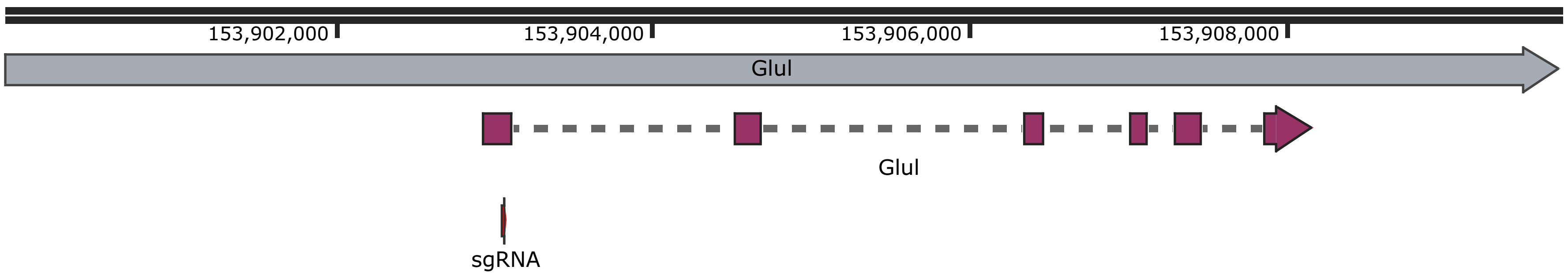

Glul
9795 bp

Created with SnapGene®

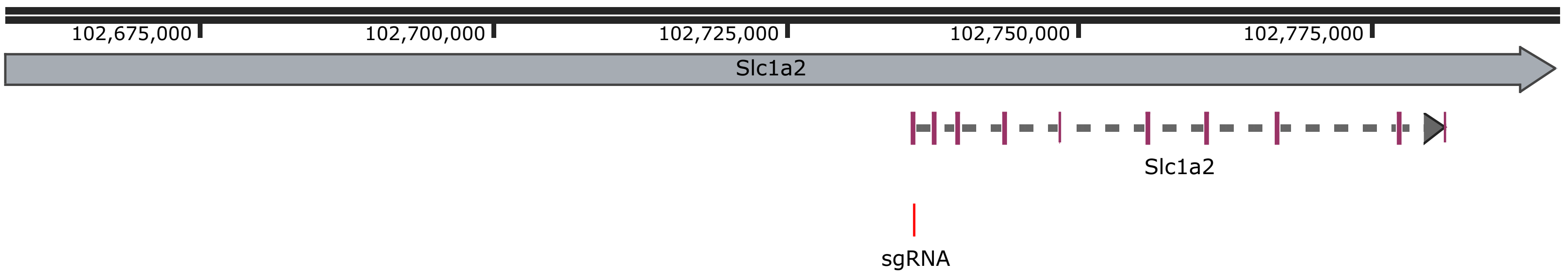

Slc1a2
132,191 bp

Created with SnapGene®

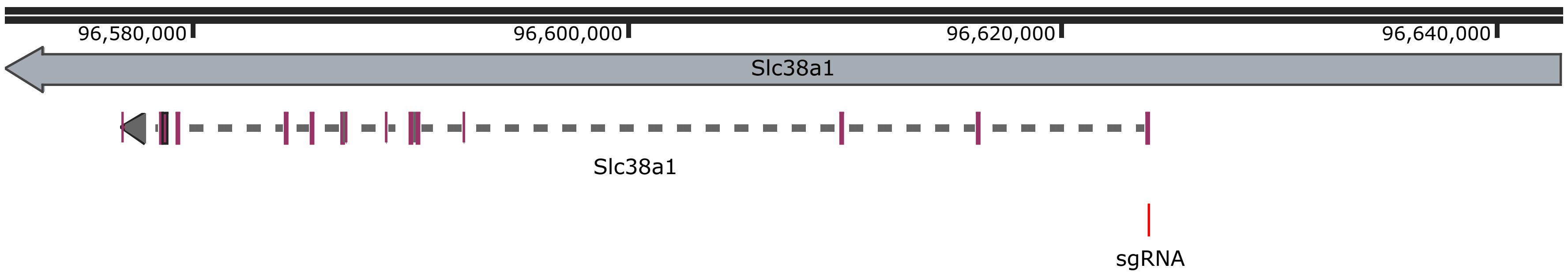

Slc38a1
71,496 bp

Created with SnapGene®

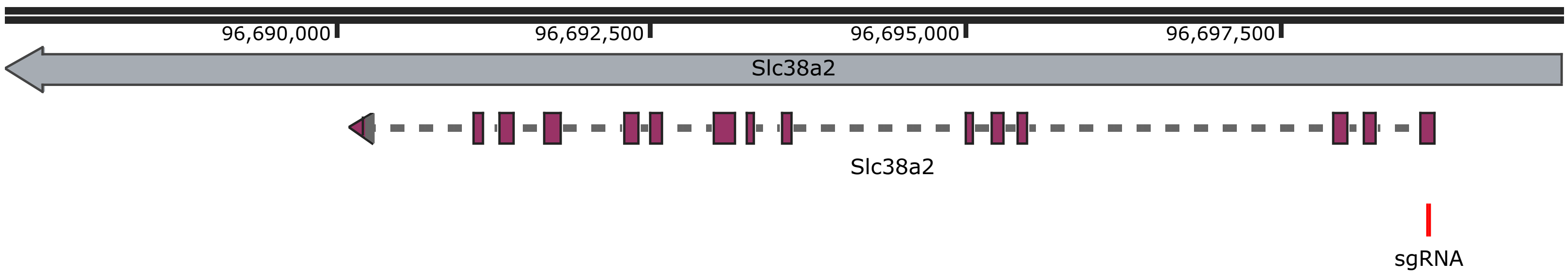

Slc38a2
12,333 bp

Created with SnapGene®

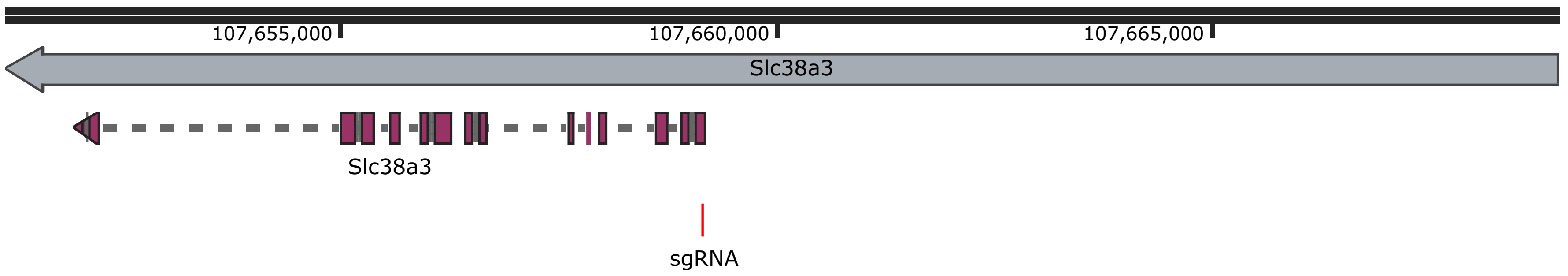

Slc38a3
17,815 bp

Created with SnapGene®

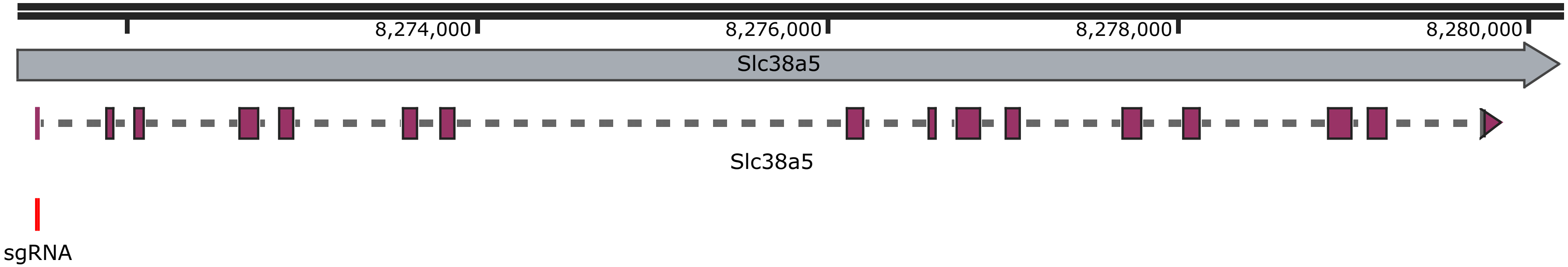

Slc38a5
8799 bp
